# Avocado Oil Supplementation Modifies Cardiovascular Risk Profile Markers in a Rat Model of Sucrose-Induced Metabolic Changes

**DOI:** 10.1155/2014/386425

**Published:** 2014-02-25

**Authors:** Octavio Carvajal-Zarrabal, Cirilo Nolasco-Hipolito, M. Guadalupe Aguilar-Uscanga, Guadalupe Melo-Santiesteban, Patricia M. Hayward-Jones, Dulce M. Barradas-Dermitz

**Affiliations:** ^1^Biochemical and Nutrition Chemistry Area, University of Veracruz, SS Juan Pablo II s/n, 94294 Boca del Río, Ver., Mexico; ^2^Department of Molecular Biology, Faculty of Resource Science and Technology, University Malaysia Sarawak, 94300 Kota Samarahan, Sarawak, Malaysia; ^3^Food Research and Development Unit, Veracruz Institute of Technology, Calz. M.A. de Quevedo 2779, 91860 Veracruz, Ver., Mexico; ^4^Pathology Laboratory, Institute of Forensic Medicine, University of Veracruz, SS Juan Pablo II s/n, 94294 Boca del Río, Ver., Mexico; ^5^Biological-Chemistry Area, Veracruz Institute of Technology, Calz. M.A. de Quevedo 2779, 91860 Veracruz, Ver., Mexico

## Abstract

The purpose of this study was to evaluate the effects of avocado oil administration on biochemical markers of cardiovascular risk profile in rats with metabolic changes induced by sucrose ingestion. Twenty-five rats were divided into five groups: a control group (CG; basic diet), a sick group (MC; basic diet plus 30% sucrose solution), and three other groups (MCao, MCac, and MCas; basic diet plus 30% sucrose solution plus olive oil and avocado oil extracted by centrifugation or using solvent, resp.). Glucose, total cholesterol, triglycerides, phospholipids, low- and high-density lipoproteins (LDL, HDL), very low-density lipoprotein (VLDL), lactic dehydrogenase, creatine kinase, and high sensitivity C-reactive protein concentration were analyzed. Avocado oil reduces TG, VLDL, and LDL levels, in the LDL case significantly so, without affecting HDL levels. An effect was exhibited by avocado oil similar to olive oil, with no significant difference between avocado oil extracted either by centrifugation or solvent in myocardial injury biochemical indicators. Avocado oil decreased hs-CRP levels, indicating that inflammatory processes were partially reversed. These findings suggested that avocado oil supplementation has a positive health outcome because it reduces inflammatory events and produces positive changes in the biochemical indicators studied, related to the development of metabolic syndrome.

## 1. Introduction

Food is a factor that plays a key role in life style, a determining influence on health and quality of life. It is known that populations with a high consumption of meat, dairy foods, and sugar have a higher mortality rate than those that feed mainly on fruits, vegetables, fish, and unsaturated oils [1]. Undesirable effects on health are associated with an excessive intake of carbohydrates (sugars) and fats. Manifestations of health disorders in people with metabolic implications are related to the incidence and prevalence of chronic and degenerative diseases such as obesity, diabetes, cardiovascular disease, and dyslipidemia (low HDL-cholesterol and high triglycerides), among others [2, 3]. Although there are many factors that contribute to its development, one of the main causes that lead to these conditions is the diet that is consumed. A diet containing a great amount of nutrients produces a strong impact on structure, physiology, and cellular metabolism. In recent years, the increase in these diseases has become a global public health problem inspite of the increasing medical knowledge for their prevention and treatment; consequently, the nutritional aspect seems to remain vital.

Statistics from the Secretary of Health in Mexico indicate that the incidence of cardiovascular diseases has increased in recent years, so that now they are the leading cause of death worldwide (WHO, 2007). On the other hand, reports found in scientific literature about the health benefits of the Mediterranean diet and olive oil have attracted interest in research on the effects and consumption of oils rich in monounsaturated fatty acids, particularly oleic acid, and its relationship with metabolic syndrome, which predisposes the individual to more serious complications, such as diabetes and cardiovascular diseases [4–6].

Mexico is a major world producer of avocados; this fruit is a rich potential source of oil (15–30 g/100 g of fruit), mostly monounsaturated [7], and a good source of linoleic acid [8]. It also contains high levels of antioxidants including polyphenols, proanthocyanidins, tocopherols, and carotenoids which have shown positive health outcomes. It has also been established that soluble components of avocado oil confer these antioxidant properties. Studies in human and animal models have shown that this oil helps to control weight, reduces the risk of diabetes [9], normalizes blood cholesterol levels [10], is involved in liver metabolism [11], and helps in skin care [12]. Other studies reported the presence of functional molecules such as glutathione [13], a molecule related to decreased risk of cancer. On the other hand, the unsaponifiable components, rich in antioxidant molecules [14], have also shown beneficial effects on anti-inflammatory processes related to the development of cancer [15].

In the relevant literature, the benefits generated by including olive oil in the diet for cardiovascular disease risk reduction are well documented. Due to similarities in lipid composition between olive oil and avocado oil, it may be assumed that the high concentration of monounsaturated fatty acids in avocado oil could be as adequate as olive oil for lowering blood lipid levels. In addition, the phytochemical components of avocado oil are also related to the disease manifestations associated with an altered metabolic profile; so overall, it is expected that all the beneficial properties of avocado oil will achieve positive health effects.

## 2. Material and Methods

### 2.1. Avocado Oil Extraction

There are different technologies for extracting oil from the avocado and they can affect its quality. The oil was obtained from Hass avocado purchased from a local market in the Port of Veracruz, Mexico. When edible maturity had been reached, the avocados were washed and peeled and the seed was removed. Subsequently, the pulp was homogenized by adding tert-butylhydroquinone (TBHQ) at 0.001% (w/w).

#### 2.1.1. Oil Extraction by Centrifugation

The avocado pulp was mixed with water to achieve a 1 : 1 w/v and NaCl (7.5% w/w), the pH was adjusted to 5.5 with ascorbic acid, and the mixture was homogenized in a blender (Black & Decker Model MX 150) at 1,300 rpm for 1 hour at 35°C. Subsequently, the oil was removed by centrifugation at 27000 rpm in a tubular continuous centrifuge (Cepa-Schnell, GLE Model NBS) fed at 2.8 L/min.

#### 2.1.2. Avocado Oil Extraction by Solvent

A homogenate was made with a portion of the avocado pulp and two parts of a mixture of hexane-isopropanol (2 : 3 v/v) in separate funnels, and the oil phase was collected. Subsequently, the solvent was removed in a rotary evaporator (Buchi R-215, Labortechnik AG, Switzerland) at 30°C and 500 mmHg pressure. The remaining solvent was removed by entrainment with nitrogen gas and then the oil was exposed to high vacuum in a freeze dryer for 24 h. Thereafter the oil was stored in refrigeration and protected from light until use.

### 2.2. Experimental Animals and Diets

In this experiment 25 male Sprague-Dawley weaned rats (6 weeks old and weighing 240 ± 16 g) were purchased from Teklad, Co. (Mexico City), and caged individually in stainless steel boxes in a room with controlled temperature (25°C) and a light-dark cycle of 12 hours. The experimental protocol for the management of experimental animals was approved by the animal ethics committee, Biochemical and Nutrition Chemistry Area, University of Veracruz. The basal diet was prepared according to the American Institute of Nutrition [16] as shown in Table 1. A mixture of corn-canola oil (7.5 g/100 g diet) was used as a source of dietary fat (Patrona from the local market). The experimental diet was prepared based on the composition of the basal diet plus oil (7.5% w/w): olive oil (carbonell), avocado oil extracted by centrifugation or solvent, respectively. Diets were prepared once a week and kept in powder form at 4°C until use. As part of this study, the fatty acid composition of the oils used in preparing diets was analyzed and it was found that all the oils had a rather similar composition, mainly oleic and linoleic acids (Table 2).

### 2.3. Sucrose-Induced Metabolic Changes Model

The animals were divided into two groups: a control group (CG, *n* = 5) receiving a basal diet and a group with sucrose-induced metabolic changes (MC, *n* = 20), which received the basal diet plus 30% sucrose solution as drinking water to induce this condition. The animals had free access to food and water for 16 weeks and food intake was measured daily. At the end of this period, the diet was withdrawn for at least 4 hours and the manifestation of the metabolic characteristics was checked by determining body weight; then serum glucose, triglycerides, and cholesterol levels were determined and obtained by cardiac puncture.

### 2.4. Animal Treatment

#### 2.4.1. Experimental Diet Management

Once the sucrose-induced metabolic changes model had been obtained, the MC animals were divided into four groups of five rats each. One group was maintained on the basal diet (the sick group, MC); three groups of rats designated, as MCao, MCac, and MCas, respectively, received an experimental diet containing 7.5% w/w oil (olive and avocado extracted by centrifugation or extracted with solvent) as the sole source of dietary fat. These four groups received the experimental diets and water with 30% sucrose solution for 4 weeks. The CG group continued to receive only the diet with corn-canola oil and no sucrose in the drinking water. Diets were prepared once a week and kept refrigerated until use. At the end of the experiment the diet was withdrawn, and the fasting animals were sacrificed through decapitation. Serum glucose, cholesterol, triglyceride, and phospholipid levels were determined. All animals were sacrificed and the organs were extracted for further analysis.

### 2.5. Biochemical Indicators

All biochemical indicator analyses were carried out on serum blood samples. Glucose was determined with the glucose oxidase method. Total cholesterol (TC), triglycerides (TG), phospholipids (PL), low- and high-density lipoprotein (LDL, HDL), very low-density lipoprotein (VLDL), lactic dehydrogenase (LDH), creatine kinase (CK), and high sensitivity C-reactive protein (hs-CRP) were determined by enzymatic colorimetric methods using commercial kits obtained from Bayer and BioMerieux, using an automated analyzer (RA 1000 XT, Bayer Technicon) and a microplate reader to determine hs-CRP. The fatty acid profile of vegetable oils was determined by gas chromatography (Hewlett Packard 5890, Palo Alto, CA.) using pentadecanoic acid as internal standard. All chemicals used were of analytical grade.

### 2.6. Statistical Analysis

The data are expressed as the mean ± standard deviation (*x* ± SD). Statistical significance was determined with analysis of variance procedures, with a post hoc Tukey multiple-range test for comparison of means (*P* < 0.05). Data were analysed using IBM^c^ SPSS^c^ Statistics Version 20, 2011.

## 3. Results

### 3.1. Metabolic Characteristics Evaluating Rats in the Control Group and Rats with Sucrose-Induced Metabolic Changes

Table 3 shows growth variables, food and caloric intake, liquid consumption and the biochemical markers assessed in rats of the control group (CG) and those with sucrose-induced metabolic changes (MC).

After 16 weeks, a significant increase (*P* < 0.05) in final body weight and body weight gain was observed in the MC group as compared to the CG group, although the food intake in rats in the CG group was significantly higher (*P* < 0.01) than in the MC group. Contrary to this, the MC group showed a daily liquid intake significantly higher (*P* < 0.05) as compared with the CG group. However, when the daily liquid intake per 100 g in weight was compared between CG and MC groups, this was not significant. The caloric equivalent produced by liquid intake was 10.8 ± 1.7 kcal in the MC group; the CG group did not have any energy intake, because this group received only purified drinking water. Triglyceride levels in the MC group were significantly greater (*P* < 0.01) than in the CG group; however, no significantly different results were found in any group for either glucose or cholesterol levels.

### 3.2. Effect of Dietary Oils on Metabolic Change Biochemical Indicators

The effect of olive and avocado oils on biochemical indicators in rats with metabolic changes induced by sucrose ingestion after the administration of experimental diets for 4 weeks is shown in Table 4.

MC group triglyceride levels increased significantly (*P* < 0.05), at least 3.7 times with respect to the CG group. On the contrary, MCao, MCac, and MCas groups exhibited reduced levels, although not significant compared to MC and not reaching the lower CG levels. As for phospholipids, the MC group showed significantly increased levels (*P* < 0.05) compared to CG; however, in MCao, MCac, and MCas groups, no significant change was observed when compared to MC, but their results were significantly higher (*P* < 0.05) when compared to CG. LDL levels in MCao and MCac groups did not show significant differences with respect to CG, but there was a very significant increase (*P* < 0.01) in the MC group, much more than the increase (*P* < 0.05) in the MCas group. VLDL in the MC group increased 3.6 times in comparison to CG levels and no significant decrease from there was observed in MCao, MCac, and MCas groups, all still significantly higher (*P* < 0.05) than CG. Significantly different results were not found for any group in the cases of glucose, cholesterol, or high-density lipoproteins (HDL).

### 3.3. Effect of Dietary Oils on Myocardial Injury Indicators

The effect of dietary olive and avocado oils on myocardial injury indicators in rats with metabolic changes induced by sucrose ingestion is shown in Table 5.

A highly significant increase (*P* < 0.01) of hs-CRP serum levels was observed in the MC group, almost double CG values. The MCao group managed to revert the change in these levels somewhat (*P* < 0.05), while MCac and MCas groups completely returned to CG values. Lactic dehydrogenase (LDH) and creatine kinase (CK) levels did not show any significant differences for any study group compared to CG; however, in MCao, MCac, and MCas groups, CK levels did fall below CG values.

## 4. Discussion

Metabolic changes are associated with a number of diseases, including obesity, diabetes, hypertension, dyslipidemia, and other abnormalities of importance related to their development. These are grouped into different profiles, such as liver, pancreatic, and cardiovascular functions.

Within this framework, in the present study, significant differences were found for MC groups as compared to the CG group in final body weight and weight gain, which were significantly higher (6 and 11%, resp.), although food intake was significantly lower (54%). These results are consistent with those reported in other studies where metabolic changes were induced by the administration of a sucrose-rich diet in addition to an experimental diet causing changes in the biochemical indicators measured [17, 18]. In relation to serum biochemical indicators associated with the development of metabolic abnormality, it was found that glucose and cholesterol concentrations in MC group rats were similar to those in the CG group and not significant. Reaven and Chang [19] have suggested that this is due to hyperinsulinemia developed in metabolic abnormalities which maintains normal levels of blood glucose. TG levels were significantly higher (56%) in MC group rats (a 2.3 fold increase). Other studies have found similar results [20, 21]; Piatti et al. [22] reported the association in healthy patients between sudden TG elevation and insulin resistance and suggested that the increase in blood TG *in vivo* inhibits glucose utilization and oxidation stimulated by insulin action in the peripheral tissues. One way to explain blood TG elevation might be to consider a possible increase in the reesterification of fatty acids from the liver as a result of fructose metabolism, as reported by Bezerra et al. [20]; this monosaccharide stems from sucrose hydrolysis and in the liver, fatty acids are mainly used for the HDL and TG synthesis, which in turn raise serum levels.

Few studies have evaluated the influence of avocado oil as a dietary fat on the lipid profile and lipoprotein metabolism, specifically in animal models with manifestations of metabolic disorders. This study found that the dietary intake of olive oil and avocado oil extracted by centrifugation or solvent exerted little or no effect on glucose, cholesterol, and HDL levels, there being no significant changes in the circulating levels of these indicators for any study group.

Among the most important effects observed in this experiment is the significant elevation of TG levels in the MC group, at least 3.8-fold compared with the CG group, a phenomenon reversed with the subsequent administration of olive oil or avocado extracted by centrifugation or solvent. This effect is attributed to the ingestion of a high amount of sucrose in the drinking water and is a feature of the metabolic disorder [23], since several studies demonstrate that high carbohydrate intake is associated with increased TG levels [24]. On the other hand, the MCao, MCac, and MCas groups were able to reduce TG levels significantly (20, 20, and 27%, resp.) compared to the MC group, although without reaching the lower levels of the CG group. These data are consistent with previous findings by Lerman-Garber et al. [9], Carranza et al. [25], and López Ledesma et al. [26], who showed that a diet supplemented with avocado oil for 30 days in diabetic subjects with induced dyslipidemia decreased TG levels by 20, 41.3, and 28.9%, respectively. In addition, the observations reported in this study add support to those postulated by Póveda et al. [27], which indicate that oils rich in monounsaturated fatty acids and micronutrients may help lower TG levels and reduce the unfavorable response in the lipid profile observed with saturated fatty acids. Phospholipids maintained a similar percentage increase (4, 2, and 0%) in the MCao, MCas, and MCac groups, with respect to the SG group, and showed no significant effect on this indicator; however, their levels were all significantly higher, by 33, 30, and 28%, respectively, in relation to the CG group. Other researchers have reported that, although olive oil has a hypocholesterolemic effect on the serum lipid profile [28], there is some evidence to suggest that it does not significantly affect the profile of heart or erythrocyte phospholipids [29]. On the other hand, it has been found that avocado oil supplementation in rats increases the phospholipid fraction in HDL as a surface component [30]; the present study shows that avocado oil, while being a crude oil high in micronutrients, also has a significant percentage of monounsaturated fatty acids (18:1N9 oleic: 54–59%), which could explain its effect to increase phospholipid levels.

It has been established that the consumption of olive oil reduces cholesterol bound to LDL (LDL-C) when replacing a source of saturated fat or one high in carbohydrates [9, 31]. This effect has been demonstrated with oils rich in monounsaturated fatty acids; however, it is not exclusive to olive oil and is also produced by other oils rich in oleic acid, such as avocado oil. This study confirmed that olive oil very significantly decreased (28%) LDL levels in the MCao group and that avocado oil extracted by centrifugation (MCac) or solvent (MCas) reduced very significantly and significantly these levels, by 26 and 23%, respectively, compared with MC group levels.

It is well known that among the relevant mechanisms of atherosclerosis pathogenesis are the oxidation of low-density lipoproteins (LDL) in the artery walls, the proliferation of smooth muscle cells, endothelial activation, and leucocyte fixation.

Among the studies linked to these mechanisms and to the presence of oleic acid are those which relate the type of fatty acid present in the oils consumed to LDL susceptibility to oxidation. Parthasarathy et al. [32] demonstrated that LDL particles rich in oleic acid are markedly more resistant to oxidative changes. This has been corroborated by Abbey et al. [33] and Reaven et al. [34] where this same particle type presented greater resistance to *ex vitro* oxidation than those rich in linoleic acid. Moreover, Mata et al. [35] reported that supplementation with monounsaturated fatty acids produces a reduction in the synthesis of smooth muscle cells in cell cultivations incubated with human serum. Studies with endothelial cells showed that oleic acid inhibits endothelial activation analysed through VCAM-1 expression (vascular cell adhesion molecule-1) [36]. Carluccio et al. [37] suggested that oleic acid contributes to atherosclerosis prevention by replacing the saturated fatty acids of cell membrane phospholipids and by modulating the genetic expression of molecules implicated in monocyte capture.

Based on all the above, it is possible to link the oleic acid present in both olive and avocado oils used in this study to the variety of mechanisms mentioned.

On the other hand, it is possible that part of the hypocholesterolemic effect observed is due to changes in the metabolism of LDL lipoproteins caused by the ingestion of avocado oil in the diet, an effect related on the one hand to the type of fat and, on the other, to the concentration of biologically active microcomponents acting additively or synergistically and not simply as isolated components [38]. Experimental data indicate that polyphenols from virgin and extra virgin oils might additionally influence lipid metabolism, thus reducing HMG-CoA reductase activity and modifying lipid values [39].

The intake of olive oil (MCao) and avocado oil extracted by centrifugation (MCac) or solvent (MCas) significantly decreased (17, 19, and 22%, resp.) the VLDL levels when compared to the SG group. On the other hand, supplementing a diet with avocado oil not only lowers LDL but also TG associated with VLDL. As it appears, in comparison with olive oil, the crude avocado oil components may be the cause of this metabolic effect in the liver, reducing triglyceride-rich lipoproteins biosynthesis. These observations have been confirmed in previous studies [40, 41].

Metabolic changes did not affect LDH levels; although the levels of this enzyme decreased (9.8%) in the MC group, their values were not significantly different compared to those in the CG group. In the olive oil group (MCao), LDH levels were reduced by 14% (3446 versus 2974 U/L), whereas in the avocado oil groups extracted by centrifugation (MCac) or solvent (MCas), LDH levels decreased by 77% and increased by 4%, respectively, compared with the MC group. Moreover, in all groups, the olive oil (MCao) and avocado oil extracted by centrifugation (MCac) or solvent (MCas) groups, LDH levels decreased by 22, 79, and 6.5%, respectively, in comparison with CG group, but no significant effect was observed because the values of this indicator overlapped with those in the CG group (2974, 802, and 3573 versus 3820 U/L, resp.).

Regarding CK, it was observed that the metabolic abnormality did not significantly affect its levels. The SG group had decreased CK levels (32%) as compared to the CG group; the olive oil group (MCao) exhibited decreased enzyme levels well below those in the CG and MC groups (55 and 35%, resp.) and a decrease of 46 and 55%, respectively, when compared to MCac and MCas data. It should be noted that although both olive oil and avocado oil groups had decreased CK levels, no significant effect was observed when compared with MC and CG groups.

Serum levels of hs-CRP in the MC group showed a very significant increase (1.76 times) compared to the CG group. This is consistent with other studies in humans, since it has been reported that hs-CRP levels are increased in subjects with signs of health disorders from metabolic abnormality [42]; this increase may also heighten the risk of cardiovascular disease [43]. Nevertheless, the olive oil group (MCao) reversed hs-CRP levels, almost reaching CG group levels (1.8 versus 1.7 U/L) but still with a 5.5% significant difference. Meanwhile, avocado oil extracted by centrifugation (MCac) or solvent (MCas) groups reduced hs-CRP levels even more than olive oil so as to attain levels statistically similar to the CG group (1.5 and 1.5 versus 1.7 mg/dL, resp.). As can be observed, both olive oil and avocado oil extracted by centrifugation or solvent reversed the metabolic changes induced by sucrose ingestion significantly and very significantly reducing hs-CRP levels by 40% in the MCao group and by 50% in the MCac and MCas groups, respectively, as compared with the MC group. It has been shown that oils rich in monounsaturated fatty acids do not increase hs-CRP levels [44]; instead, these are lowered in subjects who consume a Mediterranean diet, where the main source of monounsaturated fatty acids is olive oil [45]. Other studies show that elevated hs-CRP levels are directly related to infectious processes, inflammatory response, steatosis, cardiovascular disease, prevalence, and risk of arteriosclerotic ventricular thrombosis [42, 46–48]; this is why the use of hs-CRP has been proposed in prognostic stratification in subjects having health disorders with metabolic abnormalities [49].

The inflammatory response and its relationship with atherosclerosis-cardiovascular risk is well demonstrated; however, it is still under discussion if the measurement of increased levels of hs-CRP consistently and significantly predict cardiovascular risk from a clinical point of view [50]. In the present case, one possible explanation of a decrease in hs-CRP (an inflammation biomarker) in a diet with olive or avocado oil (obtained by any method) could be related to cytokine inhibition observed in diets with a high oleic acid content [51], considering that interleukin 6 sets off hepatocyte hs-CRP synthesis [52].

To the best of our knowledge, these markers have not been evaluated in rat models where metabolic changes induced by sucrose ingestion are associated with liver damage caused by abnormalities in liver function. A concentration of normally metabolized molecules occurs which can have a detrimental effect on health, hence the importance of these findings in the study of the effect of dietary oils such as from avocados.

In conclusion, the results suggest that avocado oil and its antioxidant content place it as a potential oil to be used as one of the preventive factors of metabolic syndrome since it reduces TG, LDL, and VLDL levels, significantly so in the case of LDL, without affecting HDL levels. Furthermore the results indicate that avocado oil exerts effects similar to olive oil, and that the type of extraction exerts an effect on only one of the biochemical indicators analyzed. It has also been found that avocado oil extracted by centrifugation or solvent decreases hs-CRP levels, indicating that inflammatory processes have been at least partially reversed, probably because the manifestation time of metabolic change was very short. Further studies are needed to elucidate the effects on cardiovascular risk profile and inflammatory markers and establish the optimal time of avocado oil supplementation in rats with sucrose-induced metabolic changes, as well as the 18:1N9 specific action on human phospholipid fraction biosynthesis in HDL as a surface component.

## Figures and Tables

**Table 1:** Composition of basal and experimental diets formulated according to AIN-76.

Ingredients	Basal diet (g)
Cornstarch	65.8
Casein	44.0
Cellulose	4.0
Mineral mix AIN-76	8.0
Vitamin mix AIN-76	2.0
DL-methionine	0.32
Tert-butylhydroquinone	0.02
Fat^†^	10.0

^†^Corn-canola in the basal diet (CG and MC groups); experimental diets (MCao, MCac, and MCas resp.) were formulated with olive oil or avocado oil, extracted either by centrifugation or solvent.

**Table 2:** Fatty acid composition of dietary oils (%).

Fatty acid	Corn	Canola	Olive	Avocado^c^	Avocado^s^
16 : 0	10.0	7.5	15.0	17.0	16.0
16 : 1	0.1	0.2	1.9	8.3	6.5
18 : 0	2.4	3.3	2.4	0.5	0.5
18 : 1	39.0	32.0	59.4	54.4	58.8
18 : 2	50.0	37.0	15.4	10.2	9.6
18 : 3	2.5	7.7	0.9	0.9	0.9

Values are expressed as mean of duplicate analysis. Avocado^c^: avocado oil extracted by centrifugation; avocado^s^: avocado oil extracted by solvent.

**Table 3:** Growth parameters, food and caloric intake, liquid consumption, and biochemical markers in control (CG) and sucrose-induced metabolic changes (MC) rats.

Variables	Dietary groups	
	CG group	MC group
Initial body weight (g)	239 ± 22	242 ± 24
Final body weight (g)	445 ± 53	470 ± 38*
Body weight gain (g)	206 ± 1.8	228 ± 2.0*
Food intake (g/d)	26.1 ± 1.3	14.3 ± 1.1**
Liquid consumption (mL/d)	46.3 ± 3.3	58.1 ± 3.4*
Liquid consumption (mL/d/100 g bw)	9.3 ± 1.4	10.5 ± 0.6
kcal equivalent in drinking water	0.00	10.8 ± 1.7**
Glucose (mg/dL)	114 ± 18	130 ± 11
Cholesterol (mg/dL)	104 ± 12	101 ± 12
Triglycerides (mg/dL)	79 ± 12	179 ± 35**

Values are mean ± SD. CG group: *n* = 5; MC group: *n* = 20. *P < 0.05; **P < 0.01.

**Table 4:** Glucose- and lipid-metabolic parameters *x*  ± SD (mg/dL) in rats fed diets with different dietary oil sources during 4 weeks.

Variables	Dietary groups				
	CG	MC	MCao	MCac	MCas
Glucose	147 ± 41	158 ± 18	155 ± 38	145 ± 16	131 ± 21
Triglycerides	48 ± 11	181 ± 29*	145 ± 42*	145 ± 58*	133 ± 28*
Cholesterol	95 ± 12	91 ± 9	97 ± 11	99 ± 12	104 ± 14
Phospholipids	43 ± 4	55 ± 4*	57 ± 4*	56 ± 4*	55 ± 6*
HDL-C	18 ± 4	18 ± 3	19 ± 3	18 ± 4	20 ± 4
LDL-C	50 ± 1	69 ± 1**	50 ± 2	51 ± 1	53 ± 1*
VLDL	10 ± 2	36 ± 6*	30 ± 10*	29 ± 11*	28 ± 6*

Values are mean ± SD.

Corn-canola diet (CG group, *n* = 5); MC group: corn-canola diet plus 30% sucrose in drinking water (*n* = 5); MCao group: olive oil plus 30% sucrose in drinking water (*n* = 5); MCac group: avocado oil extracted by centrifugation plus 30% sucrose in drinking water (*n* = 5); MCas group: avocado oil extracted by solvent plus 30% sucrose in drinking water (*n* = 5).

*P < 0.05; **P < 0.01 compared to corresponding data in CG group.

**Table 5:** Profile of myocardial injury enzymes in rats fed diets with different dietary oil sources during 4 weeks.

Variables	Dietary groups				
	CG	MC	MCao	MCac	MCas
Lactic dehydrogenase (U/L)	3820 ± 955	3446 ± 1214	2974 ± 2145	802 ± 598	3573 ± 1031
Creatine kinase (U/L)	822 ± 198	556 ± 71	364 ± 220	530 ± 358	658 ± 254
High sensitivity C-reactive protein (mg/dL)	1.7 ± 0.1	3.0 ± 0.2**	1.8 ± 0.2*	1.5 ± 0.1	1.5 ± 0.1

Values are mean ± SD.

Corn-canola diet (CG group, *n* = 5); corn-canola diet plus 30% sucrose in drinking water (MC group, *n* = 5); olive oil diet plus 30% sucrose in drinking water (MCao group, *n* = 5); avocado oil diet extracted by centrifugation plus 30% sucrose in drinking water (MCac group, *n* = 5); avocado oil diet extracted by solvent plus 30% sucrose in drinking water (MCas group, *n* = 5).

*P < 0.05; **P < 0.01 versus corresponding data in CG group.
